# The effect of chronic fluoxetine administration on anxiety-like behavior and expression of 5-HT-related proteins in rats with constitutively altered 5-HT homeostasis

**DOI:** 10.1186/2050-6511-13-S1-A30

**Published:** 2012-09-17

**Authors:** Maja Kesić, Jasminka Štefulj, Gordana Mokrović, Lipa Čičin-Šain

**Affiliations:** 1 Department of Molecular Biology, Ruđer Bošković Institute, 10000 Zagreb, Croatia

## Background

Serotonin (5-HT), a monoamine neurotransmitter/neuromodulator widely distributed in the brain, plays an important role in variety of behaviors and behavioral disorders including anxiety and depression. Therapeutic effects of fluoxetine, a widely prescribed selective serotonin reuptake inhibitor (SSRI), include inhibition of 5-HT transporters (SERT) and desensitization of 5-HT_1A_ receptors which leads to the enhancement of 5-HT transmission. The patient’s ability to respond to treatment with fluoxetine (and other SSRIs) is greatly variable and genetic SERT variants, which are believed to influence serotonergic neurotransmission, might influence interindividual variability in the pharamacotherapeutic response. In our research we use Wistar-Zagreb 5HT rats, an animal model with constitutively high or low SERT activity (termed high-5HT and low-5HT subline), developed by selective breeding toward extremes of this parameter in our laboratory. In addition to differential regulation of peripheral serotonin, 5HT-sublines also displayed constituve alteration in brain 5-HT homeostasis. Thus we have demonstrated previously that animals from the high-5HT subline exhibit increased anxiety-like behaviour; however, no measurable differences in baseline functionality or expression of 5-HT_1A_ receptors between sublines were found. Here, we examined the response of 5HT-sublines to the chronic administration of fluoxetine.

## Methods

We treated male rats from high-5HT and low-5HT sublines with fluoxetine (6 mg/kg, i.p) for 27 days. Anxiety-like behavior was evaluated 24 h after the 23rd injection by means of the elevated-plus maze paradigm. The expression of SERT and 5-HT_1A_ receptors was assessed in frontal cortices, 48 h after the last injection, using RT-PCR. At the same time point we also measured cortical 5-HT levels using an ELISA assay.

## Results

Fluoxetine-induced reduction of anxiety-like behavior, measured as increased time spent in open arms, was only observed in high-5HT animals. Furthermore, chronic fluoxetine administration increased the expression of SERT in high-5HT animals and decreased it in animals from the low-5HT subline. The expression of cortical 5-HT_1A_ receptors was not affected in high-5HT animals, whereas in the low-5HT subline a reduction in expression was noted. Tissue levels of 5-HT in fluoxetine-treated animals were significantly higher in the high-5HT subline as compared to the low-5HT subline.

## Conclusions

The present data demonstrate that fluoxetine-induced changes in 5-HT regulation exhibit clear differences between hypo- and hyperserotonergic rats. These results may contribute to the better understanding of the interindividual variability in the outcome of psychotherapy with serotonin-related drugs.

